# FGF/FGFR signaling in adrenocortical development and tumorigenesis: novel potential therapeutic targets in adrenocortical carcinoma

**DOI:** 10.1007/s12020-022-03074-z

**Published:** 2022-05-18

**Authors:** Mariangela Tamburello, Barbara Altieri, Iuliu Sbiera, Sandra Sigala, Alfredo Berruti, Martin Fassnacht, Silviu Sbiera

**Affiliations:** 1 Division of Endocrinology, Department of Internal Medicine I, University Hospital, University of Würzburg, Würzburg, Germany; 2 Section of Pharmacology, Department of Molecular and Translational Medicine, University of Brescia, Brescia, Italy; 3 Oncology Unit, Department of Medical and Surgical Specialties, Radiological Sciences, and Public Health, University of Brescia and ASST Spedali Civili di Brescia, Brescia, Italy; 4 Comprehenssive Cancer Center Mainfranken, University of Würzburg, Würzburg, Germany

**Keywords:** FGF-pathway, FGFR, FGFR-inhibitors, Adrenocortical development, Adrenocortical tumors

## Abstract

FGF/FGFR signaling regulates embryogenesis, angiogenesis, tissue homeostasis and wound repair by modulating proliferation, differentiation, survival, migration and metabolism of target cells. Understandably, compelling evidence for deregulated FGF signaling in the development and progression of different types of tumors continue to emerge and FGFR inhibitors arise as potential targeted therapeutic agents, particularly in tumors harboring aberrant FGFR signaling. There is first evidence of a dual role of the FGF/FGFR system in both organogenesis and tumorigenesis, of which this review aims to provide an overview. FGF-1 and FGF-2 are expressed in the adrenal cortex and are the most powerful mitogens for adrenocortical cells. Physiologically, they are involved in development and maintenance of the adrenal gland and bind to a family of four tyrosine kinase receptors, among which FGFR1 and FGFR4 are the most strongly expressed in the adrenal cortex. The repeatedly proven overexpression of these two FGFRs also in adrenocortical cancer is thus likely a sign of their participation in proliferation and vascularization, though the exact downstream mechanisms are not yet elucidated. Thus, FGFRs potentially offer novel therapeutic targets also for adrenocortical carcinoma, a type of cancer resistant to conventional antimitotic agents.

## Introduction

### FGF/FGFR signaling

**Fig. 1:**
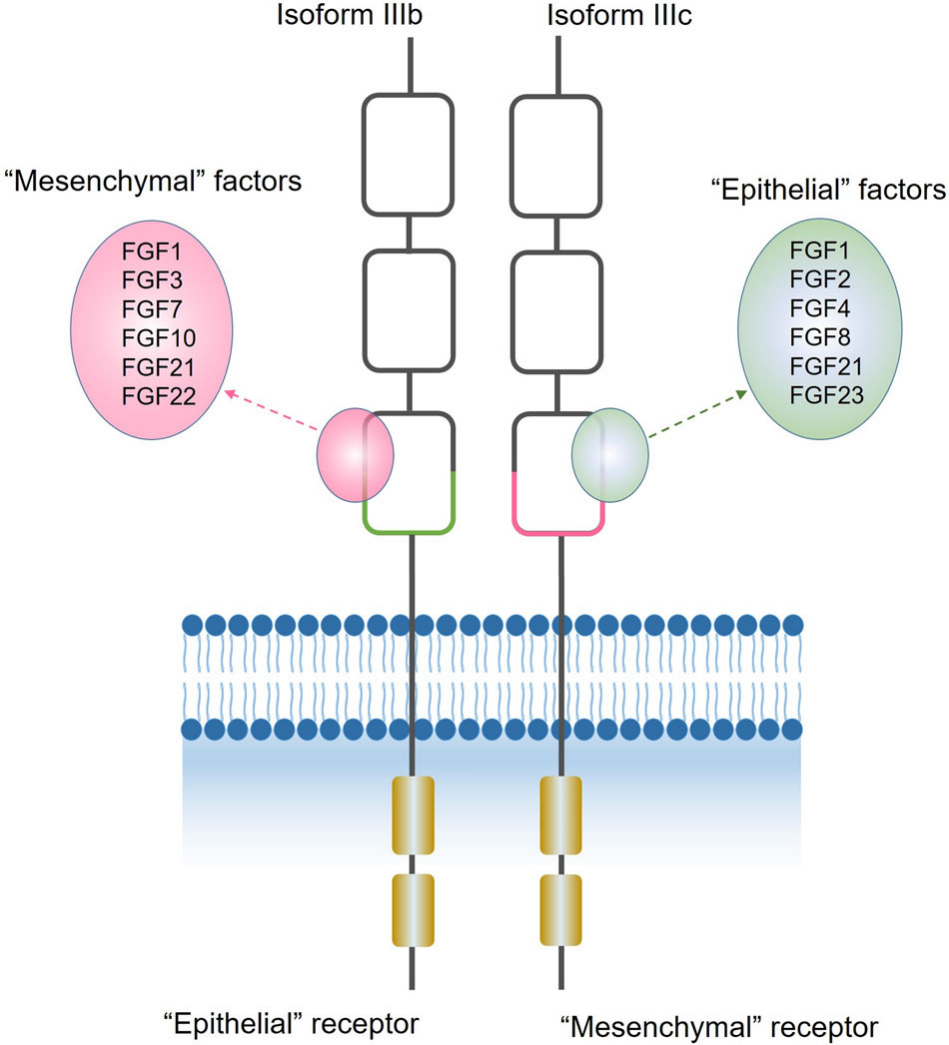
Changes in FGF to receptor binding specificity depending on tissue-specific isoform splicing for FGFR 1-3

The fibroblast growth factor (FGF)/FGF receptor (FGFR) signaling system regulates fundamental developmental pathways of multiple organ systems and plays an important role in many physiological and pathological processes in the adult organism, including the regulation of angiogenesis, tissue homeostasis, wound repair and neoplastic transformation by modulating proliferation, differentiation, survival, migration and metabolism of the cells [1–3]. The FGF family contains 22 members, usually divided into seven subfamilies according to their shared structural and functional features; eighteen among these, called canonical FGFs, are paracrine/autocrine proteins that bind and activate the tyrosine kinase (TK) receptors FGFRs, triggering an intracellular signaling cascade that mediates their biological activities [4]. The FGFR family consists of four structurally related members: FGFR 1, 2, 3 and 4, comprised of an extracellular domain, a transmembrane domain, and a split cytoplasmic TK domain [5]. The extracellular portion contains three immunoglobulin-like (Ig) folds: the IgII and IgIII domains are necessary for ligand binding while IgI and the acidic box between Ig-I and Ig-II have an auto-inhibitory function. Alternative splicing of the IgIII extracellular fragment of FGFR1 to 3 may generate isoforms that differ in terms of ligand-binding specificity, with IgIIIb and IgIIIc specifically expressed predominantly in epithelial and mesenchymal cells, respectively (Fig. 1) [6–8]. The FGF/FGFR interaction is stabilized by heparan sulfate proteoglycans (HSPGs), leading to the formation of FGF/FGFR/HSPG ternary complexes that are essential to protect FGFs from protease-mediated degradation [9]. Moreover, FGFs interact with HSPGs of the extracellular matrix, representing a reservoir of the growth factor and allowing the formation of FGF gradients that are essential for paracrine signaling [10]. After ligand binding, dimerization of the receptor causes phosphorylation of intracellular tyrosine residues that subsequently activate several signal transduction pathways [11]. Briefly, the activation of the intracellular specific adaptor protein FGFR-substrate-2 (FRS2) recruits the adaptor proteins GRB2 and SOS, activates RAS and the downstream RAF/mitogen-activated protein kinase (MAPK) pathway, mainly implicated in cell proliferation [12]. GRB2 in turn recruits GAB1, leading to the activation of the anti-apoptotic PI3K-AKT pathway [7]. Phosphorylation of phospholipase Cγ leads to the activation of protein kinase C sustains MAPK and AKT pathways and plays a role in cell migration. Depending on the cellular context, several other pathways are also activated by FGFRs including the p38 MAPK and Jun N-terminal kinase pathways, signal transducer and activator of transcription signaling and ribosomal protein S6 kinase 2 [7, 13, 14]. Negative regulation of the FGFR signaling pathway is mediated via FGF-regulated inhibitory factors such as Sprouty proteins, MAPK phosphatases MKP3 and SEF family members that modulate receptor signaling at several points in the signal transduction cascade. In addition, following activation, FGFRs are internalized and then either degraded in the proteasome or recycled according to their level of ubiquitination [6].

### FGF/FGFR signaling in adrenal development and maintenance

The pathways activated by the binding of FGFs to their receptors are critical for adrenal development and maintenance. The transcriptional coactivator CITED2 (Cbp/p300-interacting transactivator 2), which is important for the development of the adrenal glands, is regulated by basic FGF (bFGF, also known as FGF2) involving the MAPK-pathway in adrenocortical cells [15]. In the 18-day-old rat embryo [16] and human fetal adrenals [17], FGF2 is expressed in the fetal zone but appears to be absent from the definitive cortical zone. With FGF2, FGF1 and FGF9 are the only FGFs that are expressed in the embryonic adrenal gland with FGF1 mostly found in the cortex, and FGF2 and 9 preferentially in the capsule [18] (Table 1). FGFs exert mitogenic effects that are important for adrenal gland maintenance in the postnatal period [19, 20]. FGF2 stimulated proliferation of cultured fetal and definitive zone cells from midgestation [21]. FGF2 is also involved in the compensatory adrenal growth response to unilateral adrenalectomy in the rat [22]. Given the strong in vitro mitogenic activity of FGF2 on adrenocortical cells, several authors have analyzed whether it may mediate the pituitary-dependent adrenal growth. This growth was wrongly attributed for a long time to ACTH [23–26]. The discrepancy between pituitary hormone stimulation of adrenocortical cells proliferation in vivo but not in vitro could be later explained in that N-terminus proopiomelanocortin (POMC)-derived peptides (nPOMC) other than ACTH are involved in the adrenal growth stimulation [27–29]. Consistent with this theory this proliferative effect can be antagonized by ACTH, which promotes cell differentiation by inducing cell cycle arrest and steroidogenesis in vitro [21, 30–33]. Both FGF2 and nPOMC lead to proliferation through induction of ERK phosphorylation [34, 35] so it is likely that FGF2 is the mediator of nPOMC action. Moreover, FGF-2 is also a potent angiogenic and neurotrophic factor that promotes angiogenesis by directly acting on endothelial cells and by indirectly upregulating the expression of vascular endothelial growth factor (VEGF) [36, 37]. Therefore, its trophic effects could be due to the stimulation and maintenance of vascularization and innervation of the adrenal cortex in response to pituitary stimulation [38, 39]. Looking at the receptors, FGFR1 is detected at both mRNA and protein levels in the fetal cortex as well as in subpopulations of the adult cortex [17, 40]. The highest concentrations of FGFR4 were found in 17–18-week-old human fetuses’ adrenal glands. No FGFR3 was detectable in the fetal adrenal, and FGFR1 and FGFR2 mRNAs were expressed at barely detectable levels [41]. In adult adrenals, steroidogenic cells appeared to express FGFR2 and FGFR3, whereas all four receptors were detectable in the microvasculature [42]. Several reports show that the FGFR2 expression pattern in the fetal adrenal is localized to the outer cortex, where Wnt/β-catenin signaling is active [18, 43–45] (Table 1). FGFR-1 isoform IIIc and both FGFR-2 isoforms IIIb and IIIc, are expressed in both the adrenal cortex and the capsule [18, 46]. Interestingly, different from other endothelial tissues, the mesenchymal IIIc is the most expressed isoform in the adult adrenal glands [47]. The prevalence of the IIIc isoforms is related to the embryogenesis of the adrenocortical tissue, which originates from the intermediate mesoderm and undergoes a mesenchymal-epithelial transition to result in epithelial tissue. However, this epithelial transformation is incomplete, and the adrenal cortex keeps most of its mesenchymal characteristics at molecular level [47, 48]. Deletion of FGFR2, results in various degrees of adrenal hypoplasia after birth. Global knockout of isoform IIIb or both splice variants of FGFR2 results in embryonic lethality due to severe malformations [44, 49]. Recently, other authors found that mice with global deletion of the FGFR2-IIIb exhibit hypoplastic adrenals with impaired steroidogenic differentiation due to the reduced adrenal growth and impaired expression of SF1 and steroidogenic enzymes [18]. FGFR2 but not FGFR1 is required for expansion of the adrenocortical primordium by increasing proliferation and inhibiting the apoptosis in adrenocortical precursor cells [43]. Together with β-catenin, FGFR2 is also required for proper adrenal morphology by regulating cell adhesion and junction dynamics through cadherin expression modulation [45]. There is a consensus that the FGF/FGFR pathway plays a crucial role in organogenesis [50] however, there is no general principle that applies to all tissues; as seen in this chapter, in the adrenal the downstream mechanisms of this regulation have not been yet analyzed in as much detail as in other organs, future studies at molecular level is direly needed.

**Table 1:**
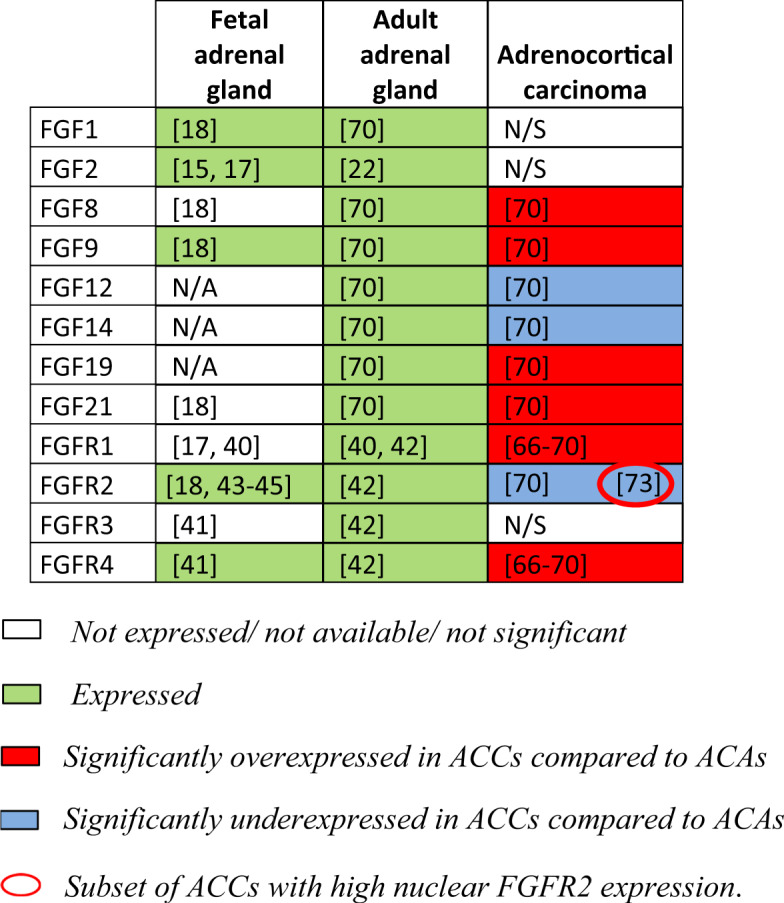
Overview of expression of select members of the FGF/FGFR pathway presented in this work in different adrenocortical tissues.

### FGFR dysregulation in cancer

There is compelling evidence that atypical regulation of the FGF/FGFR system occurs in human tumors due to various genetic alterations affecting different members of the FGF or FGFR families. This includes aberrant expression, amplifications, mutations, translocations, and fusions, leading to the deregulated activation of ligand-dependent or ligand-independent FGFR signaling [2, 51]. FGF gene abnormalities (predominantly gene amplifications) have been detected in ~14% of human malignancies [52] and a next-generation sequencing study that evaluated 4853 different tumor specimens showed that FGFR aberrations were identified in 7.1% of the tumors analyzed [53]. Gene amplifications were the most identified aberrations, present in 66% of the samples, often reported in FGFR1 and FGFR4 with variable frequencies among the different cancer types; fusions were predominantly seen in FGFR2 and FGFR3 [53, 54]. When FGFRs are mutated or amplified, aberrant activation of downstream pathways results in mitogenic and antiapoptotic responses in cells. FGF ligands are also known to promote tumor growth and proliferation by inducing neo-angiogenesis [55] through indirectly synergizing VEGF and platelet-derived growth factor pathways [56]. Some cancer models have highlighted other potential mechanisms, where FGFs might contribute to the history of a tumor such as conferring or repressing its ability to escape the restraints of the local tissue microenvironment (i.e., progress from precancerous hyperplasia to confirmed neoplasia) [57]. Furthermore, preclinical studies demonstrated FGFR crosstalk with other cell surface receptors such as G-protein-coupled receptors or other receptor TKs such as epidermal growth factor receptor, opening doors for possible therapeutic interventions with combination therapies [58]. This possibility was also confirmed in vitro by a combination of knockdown and selective pharmacological inhibition studies [2].

### Alteration of FGF/FGFR cascade in ACC

Analyses of mutations [59] and mRNA expression [60, 61] patterns in adrenocortical carcinoma (ACC) have identified disturbances in FGFR cascade with a range of 0% (for FGFR1) to 6% (for FGFR4) of cases [62]. Single nucleotide polymorphism array profiling of adrenocortical tumors has identified over 160 known oncogenes including FGFR1-3 [63]. Amplifications of FGFR1, FGF9, or FRS2 were discovered in 3 out 28 (10.7%) tumors of patients with ENSAT tumor stage III–IV by comparative genomic hybridization [64]. Giordano et al. showed that FGFR1 is among the most differentially expressed genes in ACC [65]. Using genome-wide expression studies, FGFR4 overexpression has been observed in adult but particularly in pediatric adrenocortical tumors (ACT) [66–68] (Table 1). However, the molecular mechanisms responsible for FGFR4 upregulation in ACTs have been assessed only later by Brito et al. [69]. The authors not only confirmed the previous observations by demonstrating FGFR4 overexpression in a significant proportion of pediatric (88%) and adult (47%) ACTs, but also detected FGFR4 amplifications in 13.5% of the pediatric and 30.4% of adult ACTs, suggesting that gene amplification could be the cause of FGFR4 overexpression, at least in a subset of tumors [69]. Moreover, in line with previous data [66], they found a positive correlation between FGFR4 and IGF2 expression levels, suggesting that FGFR4 and IGF2 belong to a cluster of genes that are simultaneously overexpressed in ACC. Recently, our group published the results of 93 FGF pathway-related genes in a large cohort of benign and malignant adrenocortical tissues, non-adrenal tissues and cell lines [70] (Table 1). Among the 11 genes expressed at lower levels in ACC compared to adrenocortical adenomas (ACAs), there were FGF12, FGF14, and FGFR2. The five genes significantly upregulated in ACCs vs ACAs encoded for the FGFR1, FGFR4, FGF8, and FGF19. FGF21 was the only analyzed gene that was expressed at significantly higher levels in advanced ACC. The expression of FGFRs was confirmed in a larger cohort of FFPE tissues using RNA in situ hybridization and correlated with clinical data confirming that FGFR1 and 4 were overexpressed in ACC compared to ACA, while FGFR2 was higher expressed in ACA. Moreover, a higher expression of FGFR4 was found in late ENSAT stages compared to early-stage and in recurrences/metastases compared to primary tumors [70]. Both FGFR1 and FGFR4 overexpression were significantly associated with worst prognosis [69, 70]. Some authors hypothesized that also FGFR2 expression may play a role in ACC since it regulates the differentiation and the spatial organization of the adrenal gland and it has also been linked to the activation of the Wnt/beta-catenin pathway [71], a key mechanism of adrenocortical tumorigenesis [72]. A pilot study in 26 ACCs analyzed CTNNB1 mutation status and FGFR2 expression in the same samples. The most striking result was a subset of tumors with high nuclear FGFR2 expression. However, although most tumors with the higher nuclear FGFR2 expression did not harbor a CTNNB1 mutation, the authors did not find a statistically significant association between FGFR2 expression and the mutational status of CTNNB1 or distinct clinical features [73]. Despite evidence of upregulated FGFR expression in different tumor types, it remains unclear whether such abnormal receptor expression represents the underlying molecular cause as a driver of cancer, or simply exists as a bystander, or “passenger”, event within the overall mutational profile of cancer [74].

Similar to the situation in adrenal organogenesis the knowledge is sparse and fragmented, further studies are needed to uncover the underlying mechanisms responsible for the observed effects of FGF/FGFR on adrenal tumorigenesis.

### FGFR inhibitors

FGFR inhibitors emerge more often as potential targeted therapeutic agents [75]. Particularly, preclinical studies suggest that patients presenting genomic alterations are more likely to be sensitive to FGFR inhibitors [76, 77]. Pemigatinib (PEMAZYRE^™^) and Infigratinib (TRUSELTIQ^™^) received approval by FDA for the treatment of advanced unresectable cholangiocarcinoma harboring FGFR2 fusions or rearrangement, and Erdafitinib (Balversa) for treatment of metastatic urothelial carcinoma with FGFR2 and FGFR3 genetic aberrations [58]. The preliminary molecular results would support the use of selective FGFR inhibitors also for treatment of ACC but, as of yet, these kinases have not often been targeted in dedicated trials [78]. A phase II trial including 17 patients with unresectable ACC, investigated the efficacy of dovitinib, a multi-kinase inhibitor with nonselective activity against the FGFR (targeting also colony-stimulating factor 1 receptor/CSF1R and VEGF) [79], reported only one partial response. However, 23% of patients achieved stable disease lasting longer than 6 months. A phase 1/2 study (NCT01752920) of another pan-FGFR inhibitor, ARQ087, included one ACC patient with FGFR1 gene amplification who experienced disease stabilization for 3.5 years with a maximum tumor reduction of 20% post-treatment [80]. Notably, FGFR inhibitors elicit their antitumoral effects not only directly on the cancer cells, but also indirectly through paracrine signaling blockade. Moreover, the simultaneous inhibition of FGF and CSF1 or VEGF signaling should enhance the antitumoral effects through targeting also potential immune evasion and angiogenesis in the tumor microenvironment [81] so multi-pronged therapy strategies directed at several targets would probably improve the modest results obtained until now.

## Conclusions

FGF/FGFR signaling regulates the development of the adrenal gland, enhances proliferation in adrenocortical cells and, if deregulated, can be regarded as a driver in the formation of many cancer types, including ACC. Several studies have indeed demonstrated that particularly FGFR 1 and 4 were upregulated in malignant compared to benign ACTs and that their high expression was significantly associated with worse patient prognosis, suggesting that they are potentially interesting therapeutic targets. The knowledge about FGF/FGFR signaling in adrenocortical tissues is sparse and fragmented due to a lack of studies on the molecular mechanisms. Taking inspiration from other studies performed for other organs and cancers, it appears mandatory to further investigate the underlying mechanisms of the FGF/FGFR effects in the healthy and diseased adrenal. Not only at the expression level but also looking at FGF/FGFR genetic alterations to better stratify ACC for the use of FGFR inhibitors in future clinical trials and to develop effective combinations of FGF/FGFR inhibitors with other therapies [82]. Thus, the challenge for the future is to be able to select the right patients for the most suitable targeted FGFR inhibitors therapy [76].

## References

[1] Thisse B, Thisse C (2005). Functions and regulations of fibroblast growth factor signaling during embryonic development. Dev. Biol..

[2] Turner N, Grose R (2010). Fibroblast growth factor signaling: from development to cancer. Nat. Rev. Cancer.

[3] Korc M, Friesel RE (2009). The role of fibroblast growth factors in tumor growth. Curr. Cancer Drug Targets.

[4] Itoh N, Ornitz DM (2004). Evolution of the Fgf and Fgfr gene families. Trends Genet.

[5] Farrell B, Breeze AL (2018). Structure, activation and dysregulation of fibroblast growth factor receptor kinases: perspectives for clinical targeting. Biochem. Soc. Trans..

[6] Dienstmann R, Rodon J, Prat A, Perez-Garcia J, Adamo B, Felip E (2014). Genomic aberrations in the FGFR pathway: opportunities for targeted therapies in solid tumors. Ann. Oncol..

[7] Ornitz DM, Itoh N (2015). The Fibroblast Growth Factor signaling pathway. Wiley Interdiscip. Rev. Dev. Biol..

[8] Eswarakumar VP, Lax I, Schlessinger J (2005). Cellular signaling by fibroblast growth factor receptors. Cytokine Growth Factor Rev..

[9] Schlessinger J, Plotnikov AN, Ibrahimi OA, Eliseenkova AV, Yeh BK, Yayon A (2000). Crystal structure of a ternary FGF-FGFR-heparin complex reveals a dual role for heparin in FGFR binding and dimerization. Mol. Cell.

[10] Ornitz DM (2000). FGFs, heparan sulfate and FGFRs: complex interactions essential for development. Bioessays.

[11] Schlessinger J (2000). Cell signaling by receptor tyrosine kinases. Cell.

[12] Hadari YR, Gotoh N, Kouhara H, Lax I, Schlessinger J (2001). Critical role for the docking-protein FRS2 alpha in FGF receptor-mediated signal transduction pathways. Proc. Natl Acad. Sci. USA.

[13] Goetz R, Mohammadi M (2013). Exploring mechanisms of FGF signalling through the lens of structural biology. Nat. Rev. Mol. Cell Biol..

[14] Presta M, Chiodelli P, Giacomini A, Rusnati M, Ronca R (2017). Fibroblast growth factors (FGFs) in cancer: FGF traps as a new therapeutic approach. Pharm. Ther..

[15] Haase M, Schott M, Bornstein SR, Malendowicz LK, Scherbaum WA, Willenberg HS (2007). CITED2 is expressed in human adrenocortical cells and regulated by basic fibroblast growth factor. J. Endocrinol..

[16] Gonzalez AM, Buscaglia M, Ong M, Baird A (1990). Distribution of basic fibroblast growth factor in the 18-day rat fetus: localization in the basement membranes of diverse tissues. J. Cell Biol..

[17] Gonzalez AM, Hill DJ, Logan A, Maher PA, Baird A (1996). Distribution of fibroblast growth factor (FGF)-2 and FGF receptor-1 messenger RNA expression and protein presence in the mid-trimester human fetus. Pediatr. Res..

[18] Guasti L, Candy Sze WC, McKay T, Grose R, King PJ (2013). FGF signalling through Fgfr2 isoform IIIb regulates adrenal cortex development. Mol. Cell Endocrinol..

[19] Boulle N, Gicquel C, Logié A, Christol R, Feige JJ, Le Bouc Y (2000). Fibroblast growth factor-2 inhibits the maturation of pro-insulin-like growth factor-II (Pro-IGF-II) and the expression of insulin-like growth factor binding protein-2 (IGFBP-2) in the human adrenocortical tumor cell line NCI-H295R. Endocrinology.

[20] Feige JJ, Vilgrain I, Brand C, Bailly S, Souchelnitskiy S (1998). Fine tuning of adrenocortical functions by locally produced growth factors. J. Endocrinol..

[21] Crickard K, Ill CR, Jaffe RB (1981). Control of proliferation of human fetal adrenal cells in vitro. J. Clin. Endocrinol. Metab..

[22] Basile DP, Holzwarth MA (1993). Basic fibroblast growth factor may mediate proliferation in the compensatory adrenal growth response. Am. J. Physiol..

[23] Hornsby PJ, Gill GN (1977). Hormonal control of adrenocortical cell proliferation. Desensitization to ACTH and interaction between ACTH and fibroblast growth factor in bovine adrenocortical cell cultures. J. Clin. Investig..

[24] Savona C, Feige JJ (1991). cAMP-mediated regulation of adrenocortical cell bFGF receptors. Ann. N. Y Acad. Sci..

[25] Ishimoto H, Jaffe RB (2011). Development and function of the human fetal adrenal cortex: a key component in the feto-placental unit. Endocr. Rev..

[26] Mesiano S, Jaffe RB (1997). Role of growth factors in the developmental regulation of the human fetal adrenal cortex. Steroids.

[27] Estivariz FE, Carino M, Lowry PJ, Jackson S (1988). Further evidence that N-terminal pro-opiomelanocortin peptides are involved in adrenal mitogenesis. J. Endocrinol..

[28] Estivariz FE, Morano MI, Carino M, Jackson S, Lowry PJ (1988). Adrenal regeneration in the rat is mediated by mitogenic N-terminal pro-opiomelanocortin peptides generated by changes in precursor processing in the anterior pituitary. J. Endocrinol..

[29] Fassnacht M, Hahner S, Hansen IA, Kreutzberger T, Zink M, Adermann K (2003). N-Terminal Proopiomelanocortin Acts as a Mitogen in Adrenocortical Tumor Cells and Decreases Adrenal Steroidogenesis. J. Clin. Endocrinol. Metab..

[30] Chu Y, Ho WJ, Dunn JC (2009). Basic fibroblast growth factor delivery enhances adrenal cortical cellular regeneration. Tissue Eng. Part A.

[31] Gospodarowicz D, Handley HH (1975). Stimulation of division of Y1 adrenal cells by a growth factor isolated from bovine pituitary glands. Endocrinology.

[32] Gospodarowicz D, Ill CR, Hornsby PJ, Gill GN (1977). Control of bovine adrenal cortical cell proliferation by fibroblast growth factor. Lack of effect of epidermal growth factor. Endocrinology.

[33] Lepique AP, Moraes MS, Rocha KM, Eichler CB, Hajj GN, Schwindt TT (2004). c-Myc protein is stabilized by fibroblast growth factor 2 and destabilized by ACTH to control cell cycle in mouse Y1 adrenocortical cells. J. Mol. Endocrinol..

[34] Mattos GE, Jacysyn JF, Amarante-Mendes GP, Lotfi CF (2011). Comparative effect of FGF2, synthetic peptides 1-28 N-POMC and ACTH on proliferation in rat adrenal cell primary cultures. Cell Tissue Res..

[35] Lotfi CF, Costa ET, Schwindt TT, Armelin HA (2000). Role of ERK/MAP kinase in mitogenic interaction between ACTH and FGF2 in mouse Y1 adrenocortical tumor cells. Endocr. Res..

[36] Bikfalvi A, Klein S, Pintucci G, Rifkin DB (1997). Biological roles of fibroblast growth factor-2. Endocr. Rev..

[37] Presta M, Dell’Era P, Mitola S, Moroni E, Ronca R, Rusnati M (2005). Fibroblast growth factor/fibroblast growth factor receptor system in angiogenesis. Cytokine Growth Factor Rev..

[38] Gospodarowicz D, Cheng J, Lui GM, Baird A, Esch F, Bohlen P (1985). Corpus luteum angiogenic factor is related to fibroblast growth factor. Endocrinology.

[39] Mesiano S, Jaffe RB (1997). Developmental and functional biology of the primate fetal adrenal cortex. Endocr. Rev..

[40] Meisinger C, Hertenstein A, Grothe C (1996). Fibroblast growth factor receptor 1 in the adrenal gland and PC12 cells: developmental expression and regulation by extrinsic molecules. Brain Res. Mol. Brain Res..

[41] Partanen J, Mäkelä TP, Eerola E, Korhonen J, Hirvonen H, Claesson-Welsh L (1991). FGFR-4, a novel acidic fibroblast growth factor receptor with a distinct expression pattern. Embo J..

[42] Hughes SE (1997). Differential expression of the fibroblast growth factor receptor (FGFR) multigene family in normal human adult tissues. J. Histochem. Cytochem..

[43] Häfner R, Bohnenpoll T, Rudat C, Schultheiss TM, Kispert A (2015). Fgfr2 is required for the expansion of the early adrenocortical primordium. Mol. Cell Endocrinol..

[44] Kim AC, Hammer GD (2007). Adrenocortical cells with stem/progenitor cell properties: recent advances. Mol. Cell Endocrinol..

[45] Leng S, Pignatti E, Khetani RS, Shah MS, Xu S, Miao J (2020). β-Catenin and FGFR2 regulate postnatal rosette-based adrenocortical morphogenesis. Nat. Commun..

[46] Walczak EM, Hammer GD (2015). Regulation of the adrenocortical stem cell niche: implications for disease. Nat. Rev. Endocrinol..

[47] I. Sbiera, S. Kircher, B. Altieri, M. Fassnacht, M. Kroiss, S. Sbiera, Epithelial and Mesenchymal Markers in Adrenocortical Tissues: How Mesenchymal Are Adrenocortical Tissues? Cancers (Basel).13(7), (2021). 10.3390/cancers13071736. 10.3390/cancers13071736 PMC8038668 33917436

[48] Xing Y, Lerario AM, Rainey W, Hammer GD (2015). Development of adrenal cortex zonation. Endocrinol. Metab. Clin. North Am..

[49] Revest JM, Spencer-Dene B, Kerr K, De Moerlooze L, Rosewell I, Dickson C (2001). Fibroblast growth factor receptor 2-IIIb acts upstream of Shh and Fgf4 and is required for limb bud maintenance but not for the induction of Fgf8, Fgf10, Msx1, or Bmp4. Dev. Biol..

[50] M.E. Pownall, H. V. Isaacs, Developmental Biology. *FGF Signalling in Vertebrate Development.* (Morgan & Claypool Life Sciences. Copyright © 2010 by Morgan & Claypool Life Sciences, San Rafael (CA), 2010). 21452439

[51] Krook MA, Reeser JW, Ernst G, Barker H, Wilberding M, Li G (2021). Fibroblast growth factor receptors in cancer: genetic alterations, diagnostics, therapeutic targets and mechanisms of resistance. Br. J. Cancer.

[52] Helsten T, Schwaederle M, Kurzrock R (2015). Fibroblast growth factor receptor signaling in hereditary and neoplastic disease: biologic and clinical implications. Cancer Metastasis Rev..

[53] Helsten T, Elkin S, Arthur E, Tomson BN, Carter J, Kurzrock R (2016). The FGFR Landscape in Cancer: analysis of 4853 Tumors by Next-Generation Sequencing. Clin. Cancer Res..

[54] Chen L, Zhang Y, Yin L, Cai B, Huang P, Li X (2021). Fibroblast growth factor receptor fusions in cancer: opportunities and challenges. J. Exp. Clin. Cancer Res..

[55] Ronca R, Giacomini A, Rusnati M, Presta M (2015). The potential of fibroblast growth factor/fibroblast growth factor receptor signaling as a therapeutic target in tumor angiogenesis. Expert Opin. Ther. Targets.

[56] Lieu C, Heymach J, Overman M, Tran H, Kopetz S (2011). Beyond VEGF: inhibition of the fibroblast growth factor pathway and antiangiogenesis. Clin. Cancer Res..

[57] Grose R, Dickson C (2005). Fibroblast growth factor signaling in tumorigenesis. Cytokine Growth Factor Rev..

[58] A. Kommalapati, S.H. Tella, M. Borad, M. Javle, A. Mahipal, FGFR Inhibitors in Oncology: Insight on the Management of Toxicities in Clinical Practice. Cancers (Basel).13(12), (2021). 10.3390/cancers13122968. 10.3390/cancers13122968 PMC8231807 34199304

[59] Zheng S, Cherniack AD, Dewal N, Moffitt RA, Danilova L, Murray BA (2016). Comprehensive Pan-Genomic Characterization of Adrenocortical Carcinoma. Cancer Cell.

[60] Assie G, Giordano TJ, Bertherat J (2012). Gene expression profiling in adrenocortical neoplasia. Mol. Cell Endocrinol..

[61] de Reyniès A, Assié G, Rickman DS, Tissier F, Groussin L, René-Corail F (2009). Gene expression profiling reveals a new classification of adrenocortical tumors and identifies molecular predictors of malignancy and survival. J. Clin. Oncol..

[62] Altieri B, Ronchi CL, Kroiss M, Fassnacht M (2020). Next-generation therapies for adrenocortical carcinoma. Best. Pr. Res Clin. Endocrinol. Metab..

[63] Ronchi CL, Sbiera S, Leich E, Henzel K, Rosenwald A, Allolio B (2013). Single nucleotide polymorphism array profiling of adrenocortical tumors–evidence for an adenoma carcinoma sequence?. PLoS ONE.

[64] De Martino MC, Al Ghuzlan A, Aubert S, Assié G, Scoazec JY, Leboulleux S (2013). Molecular screening for a personalized treatment approach in advanced adrenocortical cancer. J. Clin. Endocrinol. Metab..

[65] Giordano TJ, Thomas DG, Kuick R, Lizyness M, Misek DE, Smith AL (2003). Distinct transcriptional profiles of adrenocortical tumors uncovered by DNA microarray analysis. Am. J. Pathol..

[66] de Fraipont F, El Atifi M, Cherradi N, Le Moigne G, Defaye G, Houlgatte R (2005). Gene expression profiling of human adrenocortical tumors using complementary deoxyribonucleic Acid microarrays identifies several candidate genes as markers of malignancy. J. Clin. Endocrinol. Metab..

[67] Laurell C, Velázquez-Fernández D, Lindsten K, Juhlin C, Enberg U, Geli J (2009). Transcriptional profiling enables molecular classification of adrenocortical tumours. Eur. J. Endocrinol..

[68] West AN, Neale GA, Pounds S, Figueredo BC, Rodriguez Galindo C, Pianovski MA (2007). Gene expression profiling of childhood adrenocortical tumors. Cancer Res..

[69] Brito LP, Ribeiro TC, Almeida MQ, Jorge AA, Soares IC, Latronico AC (2012). The role of fibroblast growth factor receptor 4 overexpression and gene amplification as prognostic markers in pediatric and adult adrenocortical tumors. Endocr. Relat. Cancer.

[70] Sbiera I, Kircher S, Altieri B, Lenz K, Hantel C, Fassnacht M (2021). Role of FGF Receptors and Their Pathways in Adrenocortical Tumors and Possible Therapeutic Implications. Front. Endocrinol. (Lausanne).

[71] Krejci P, Aklian A, Kaucka M, Sevcikova E, Prochazkova J, Masek JK (2012). Receptor tyrosine kinases activate canonical WNT/β-catenin signaling via MAP kinase/LRP6 pathway and direct β-catenin phosphorylation. PLoS ONE.

[72] Juhlin CC, Goh G, Healy JM, Fonseca AL, Scholl UI, Stenman A (2015). Whole-Exome Sequencing Characterizes the Landscape of Somatic Mutations and Copy Number Alterations in Adrenocortical Carcinoma. J. Clin. Endocrinol. Metab..

[73] Haase M, Thiel A, Scholl UI, Ashmawy H, Schott M, Ehlers M (2020). Subcellular localization of fibroblast growth factor receptor type 2 and correlation with CTNNB1 genotype in adrenocortical carcinoma. BMC Res. Notes.

[74] Ahmad I, Iwata T, Leung HY (2012). Mechanisms of FGFR-mediated carcinogenesis. Biochim. Biophys. Acta.

[75] Giacomini A, Chiodelli P, Matarazzo S, Rusnati M, Presta M, Ronca R (2016). Blocking the FGF/FGFR system as a “two-compartment” antiangiogenic/antitumor approach in cancer therapy. Pharm. Res..

[76] Dieci MV, Arnedos M, Andre F, Soria JC (2013). Fibroblast growth factor receptor inhibitors as a cancer treatment: from a biologic rationale to medical perspectives. Cancer Disco..

[77] Chae YK, Ranganath K, Hammerman PS, Vaklavas C, Mohindra N, Kalyan A (2017). Inhibition of the fibroblast growth factor receptor (FGFR) pathway: the current landscape and barriers to clinical application. Oncotarget.

[78] Konda B, Kirschner LS (2016). Novel targeted therapies in adrenocortical carcinoma. Curr. Opin. Endocrinol. Diabetes Obes..

[79] J. García-Donas, S. Hernando Polo, M. Guix, M.A. Climent Duran, M.J. Méndez-Vidal, P. Jiménez-Fonseca, et al. Phase II study of dovitinib in first line metastatic or (non resectable primary) adrenocortical carcinoma (ACC): SOGUG study 2011-03. Journal of Clinical Oncology 32(15_suppl):4588-4588 (2014). 10.1200/jco.2014.32.15_suppl.4588

[80] Papadopoulos KP, El-Rayes BF, Tolcher AW, Patnaik A, Rasco DW, Harvey RD (2017). A Phase 1 study of ARQ 087, an oral pan-FGFR inhibitor in patients with advanced solid tumours. Br. J. Cancer.

[81] Katoh M (2016). FGFR inhibitors: Effects on cancer cells, tumor microenvironment and whole-body homeostasis (Review). Int J. Mol. Med..

[82] Ghedini GC, Ronca R, Presta M, Giacomini A (2018). Future applications of FGF/FGFR inhibitors in cancer. Expert Rev. Anticancer Ther..

